# Characteristics and outcomes of fetal ventricular aneurysm and diverticulum: combining the use of a new technique, fetal HQ

**DOI:** 10.3389/fped.2023.1165972

**Published:** 2023-05-04

**Authors:** Liqing Zhao, Pengfei Wu, Xianting Jiao, Minjie Zhang, Wenhao Jing, Yurong Wu, Sun Chen

**Affiliations:** Department of Pediatric Cardiology, Xinhua Hospital Affiliated to Shanghai Jiao Tong University School of Medicine, Shanghai, China

**Keywords:** ventricular aneurysm, ventricular diverticulum, fetal echocardiography, fetal heart, sphericity index

## Abstract

**Objectives:**

Congenital ventricular aneurysms or diverticulum (VA/VD) are rare cardiac anomalies with lack prenatal evaluation data. The present study aimed to provide the prenatal characteristics and outcomes from a tertiary center and the use of new techniques to evaluate the shape and contractility of these fetuses.

**Methods:**

Ten fetuses were diagnosed with VA or VD, and 30 control fetuses were enrolled. Fetal echocardiography was performed to make the diagnosis. The prenatal echo characteristics and follow-up data were carefully reviewed. The shape and contractility measurements of the four-chamber view (4CV) and both ventricles were measured and computed using fetal fetal heart quantification (HQ).

**Results:**

A total of 10 fetuses were enrolled, including 4 cases of left ventricular diverticulum, 5 cases of left ventricular aneurysm, and 1 case of right ventricular aneurysm (RVA). Four cases chose to terminate the pregnancy. The RVA was associated with a perimembranous ventricular septal defect. Two cases had fetal arrhythmia, and one case had pericardial effusion. After birth, one case underwent surgical resection at five years old. The 4CV global sphericity index (SI) of free-wall located ventricular outpouching (VO) was significantly lower than the apical ones and the control group (*p* < 0.01). Four of five apical left VOs had significant higher (>95th centile) SI in base segments, and three of four left VOs in the free-wall had significant lower (< 5th centile) SI in the majority of 24 segments. Compared to the control group, the left ventricle (LV) global longitudinal strain, ejection fraction, and fractional area change were significantly decreased (*p* < 0.01), while the LV cardiac output of the cases was in the normal range. The transverse fraction shortening of the affected segments of ventricles was significantly lower than the other ventricle segments (*p* < 0.01).

**Conclusions:**

Fetal HQ is a promising technique to evaluate the shape and contractility of congenital ventricular aneurysm and diverticulum.

## 1. Introduction

Primary congenital ventricular aneurysm (VA) and ventricular diverticulum (VD) are rare congenital cardiac malformations. Both are characterized by localized outpouchings of the ventricular wall, most frequently the left ventricle (LV) (1). The prevalence ranges from 0.02% to 0.76% according to different studies (2, 3). The different diagnosis between VA and VD is still controversial; VA cases were thought to be aneurysms with wide connections to the ventricle and had lower contractility and poor prognosis in most conditions compared to VD (4). They were also called by the joint name of “ventricular outpouching (VO)” in some research (5, 6).

Nowadays, most structural cardiac anomalies can be detected prenatally (7). The existence of VD or VA can be easily identified through the four-chamber view (4CV), especially big ones, but accurate evaluation and consultation are difficult. The clinical outcomes of VA/VD range from fetal demise to asymptomatic survival. When identified during the fetal period, it is important to give a comprehensive consultation to the pregnancy and the family. Limited data are available concerning prenatal diagnosis and consultation of VD and VA (3). This may be due to the rarity of the disease and the paucity of data using novel methods in evaluating the fetal morphology and function of fetal VA or VD.

Fetal heart morphology and contractility can be measured both global and segmental by a new technique, fetal heart quantification (HQ), a quantitative speckle-tracking analysis of both ventricular endocardium from the base to the apex. Sphericity index (SI) and fraction shortening (FS) of 24 segments are thought to be comprehensive methods in fetal heart assessment, and are independent of gestational age and fetal biometric measurements (8, 9).

The objective of the present study was to provide the prenatal characteristics and outcome of VA/VD from our fetal heart center and evaluate the added value of fetal HQ in the prenatal evaluation and diagnosis of VA/VD.

## 2. Materials and methods

### 2.1. Study population

This was a retrospective study that included pregnancies referred to the Fetal Heart Center, Xinhua hospital affiliated to Shanghai JiaoTong University School of Medicine, for extended fetal echocardiography, and fetuses that were diagnosed with congenital ventricular aneurysm or diverticulum.

The study protocol was approved by the ethics committee at the authors’ affiliated hospital (No. XHEC-QT-2021-042). Written informed consent was signed before the examination, and all pregnant women agreed to use the images obtained during the examination for study purpose.

### 2.2. Control group

Thirty fetuses without ultrasound-detected cardiac malformation were used as the control group in the present study. They were also free of growth disturbances and other detected genetic or organ abnormalities. Measurements mentioned below were also obtained from these fetuses and compared with built-in references and reported studies.

### 2.3. Fetal echo and image acquisition

**Figure 1:**
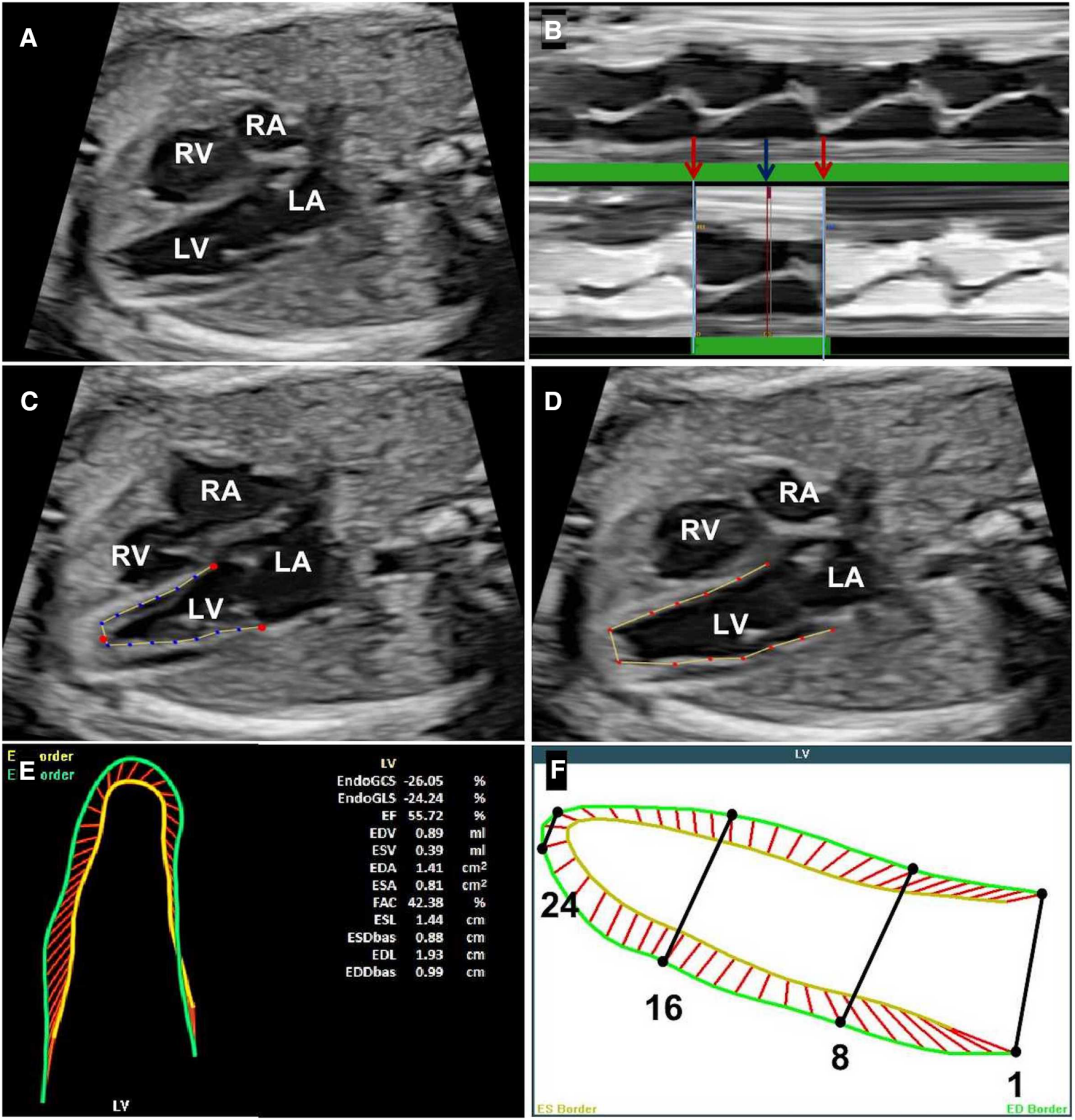
Steps for accomplishing the analysis of fetal HQ. (**A**) The four-chamber view of the fetus under analysis. (**B**) Identification of a single cardiac cycle with the help of the M-mode; the red arrows represent the end-diastolic phase and the blue arrow represents the end-systolic phase. (**C**) The end-systolic endocardial border tracing. (**D**) The end-diastolic endocardial border tracing. (**E**) The shape and contractility values calculated by fetal HQ. (**F**) 24-segment delineation from ventricular base to apex. LA, left atrium; LV, left ventricle; RA, right atrium; RV, right ventricle.

Fetal echocardiography was performed by experienced experts (YW and SC) to make the diagnosis, and two-dimensional images of the four-chamber view (4CV) (Figure 1A) were acquired using the Voluson E8 or E10 (GE Healthcare Ultrasound, WI, United States) with a 4–8 MHz transabdominal probe. Without histological information, the differential diagnoses of VA and VD depended on morphological characteristics, observed contractility, and expert experience. “VA” diagnosis was made when the outpouching was comparatively huge, with a broad neck and low contractility. Images were optimized to enhance the borders of both chamber and endocardium in the 4CV. At least 3-s cine clips of the four-chamber view were stored as uncompressed (.4dv) files in a separate online database. Voluson E10 is equipped with fetal HQ software (GE Healthcare Ultrasound, WI, United States) for further analysis.

### 2.4. Image analysis with fetal HQ

Image analysis was accomplished using fetal HQ measurements software build-in the Voluson E10 machine. All image analyses were done by one operator (LZ). Each fetus was measured three times and the intraobserver variability was calculated. The time of accomplishing the analysis of a single image was less than 5 min.

The cardiac cycle was identified by drawing an M-mode line parallel to the annulus of the RV wall. A single cardiac cycle was identified by three lines, two identify the adjacent end-diastolic phase and one tells the end-systolic phase between them (Figure 1B). Then, three dots were put according to the instruction image; the endocardial borders can be automatically detected in both the end-systolic (Figure 1C) and the end-diastolic phases (Figure 1D). Manual adjustment can be done when the automatic trace was not satisfied.

Once the endocardium trace was confirmed, measurements and associated calculations can be done automatically in a few seconds (Figure 1E). The values, centiles, and *z*-scores were measured and computed for both geometry and contractility, including 4CV end-diastolic width, 4CV end-diastolic length, 4CV end-diastolic volume, global sphericity index (GSI), 24-segment SI, 24-segment end-diastolic width, global longitudinal strain (GLS) of both ventricles, fractional area change (FAC), left ventricular ejection fraction (LV EF), LV cardiac output (CO), FS, and 24-segment transverse FS. Twenty-four segments are equally divided from base to apical. Segments 1–8 are considered base sections, segments 9–16 are middle sections, and segments 17–24 are apical sections (Figure 1F).

### 2.5. Statistical analysis

All statistical analyses were performed with the statistical software SPSS version 26 (IBM, United States). Continuous variables were expressed as mean ± standard deviation (SD) or median (interquartile range, IQR), and categorical variables were expressed in frequency. The *z*-score above +1.65 (95th centile) or below −1.65 (5th centile) was considered abnormal values. Student's *t* test and *χ*^2^ test were used to determine the differences between groups. *p*-values <0.05 were considered statistically significant. Intraclass correlation coefficients (ICCs) were assessed for intraobserver variability using a two-way random-effects model. ICC >0.80 was considered excellent, and good at 0.60–0.80.

## 3. Results

### 3.1. Baseline characteristics of the study subjects

Totally 11 fetuses were prenatally diagnosed with VD or VA from August 2015 to September 2022 in our fetal heart center from our database. One case was excluded from fetal HQ analysis because of an unsatisfied 4CV image. Basic characteristics and main findings from fetal echocardiography are listed in Table 1. The mean age of pregnant women was 31.5 ± 5.26 years, and the median gestation age of the first evaluation was 26 gestational weeks (GWs), ranging from 22 to 36 GWs. Among the 10 enrolled fetuses, there were 4 cases of left ventricular diverticulum (LVD), 5 cases of left ventricular aneurysm (LVA), and 1 case of right ventricular aneurysm (RVA). During the follow-up, four cases chose to terminate the pregnancy. The case of RVA was associated with a perimembranous ventricle septal defect (VSD). Two fetuses were complicated with fetal arrhythmia, and both presented with premature ventricular contractions (PVC). Pericardial effusion (PE) and mild mitral regurgitation existed in one fetus.

**Table 1:** Basic characteristics and fetal echocardiography findings of the enrolled cases.

Case	Type	Diagnostic GA (weeks)	Maternal age (years)	Location	Size (length × width, mm)	Size (mm^2^)	Neck diameter (mm)	Vaginal delivery/ VA area/ventricular area	CTR	Perinatal complications	Perinatal outcome	Postnatal follow-up (months)
1	LVD	23^+3^	26	Apical	9.6 × 4.7	79	9.9	0.36	0.27	—	vaginal delivery 38^+5^ GW	75
2	LVA	24^+5^	36	Apical	16 × 11.1	121	12.1	0.43	0.43	—	TOP	—
3	LVA	32^+6^	28	Free-wall	16.3 × 7	82	5.6	0.26	0.38	—	vaginal delivery 39 GW	35
4	LVA	34	39	Apical	19.5 × 7.6	205	8.7	0.49	0.32	Fetal arrhythmia	vaginal delivery 38^+3^ GW	10
5	RVA	26^+3^	32	Free-wall	8.8 × 9.9	61	5	0.29	0.31	VSD	TOP	—
6	LVA	22^+4^	28	Free-wall	17.7 × 9.9	141	7.2	0.49	0.49	PE, MR	TOP	—
7	LVD	34^+4^	29	Apical	21.9 × 16.7	280	8.5	0.5	0.43	Fetal arrhythmia	vaginal delivery 38^+6^ GW	23
8	LVD	24^+1^	24	Apical	10.9 × 10.5	80	7.5	0.39	0.31	—	vaginal delivery 38^+2^ GW	5
9	LVD	28^+6^	38	Free-wall	16.8 × 9	139	4.2	0.49	0.38	—	vaginal delivery 39^+1^ GW	1
10	LVA	26^+1^	35	Apical	15.2 × 8.5	95	9.5	0.38	0.39	—	TOP	—

VA, ventricular aneurysm; LVD, left ventricular diverticulum; LVA, left ventricular aneurysm; RVA, right ventricular aneurysm; GA, gestational age; CTR, cardiothoracic ratio; VSD, ventricular septal defect; PE, pericardial effusion; MR, mitral regurgitation; TOP, termination of pregnancy; GW, gestational week.

### 3.2. Two-dimensional echocardiography findings

**Figure 2:**
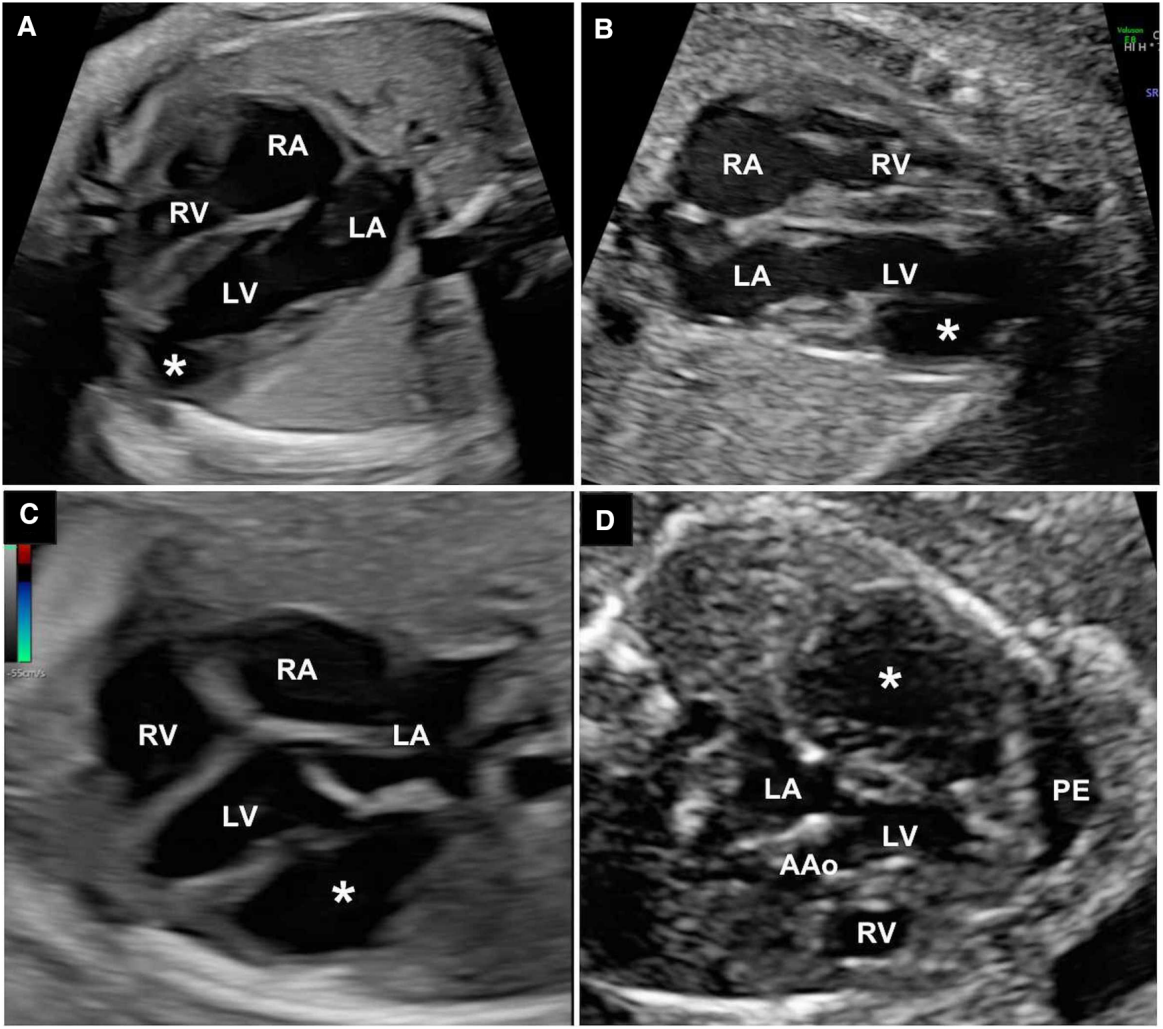
(**A**) The 4CV of apical left ventricle diverticulum. (**B**) 4CV of apical left ventricle aneurysm. (**C**) 4CV of LVA at the ventricular free-wall; (**D**) The LVA case with PE. White asterisks represent ventricle aneurysm or diverticulum. 4CV, four-chamber view; LA, left atrium; LV, left ventricle; RA, right atrium; RV, right ventricle; AAo, ascending aorta; PE, pericardial effusion; LVA, left ventricular aneurysm.

The prenatal diagnosis of VA or VD was made after a careful fetal echocardiography examination. Regardless of the size, six cases (60%, 6/10) of VA/VD were located at the ventricular apex, and the other four (40%, 4/10) were located at the free-wall of ventricles including one RVA. The mean neck diameter that connects with the ventricle was 7.82 ± 2.43 mm. The mean area of VA/VD was 128.3 ± 68.13 mm^2^, and the mean ratio compared to the affected ventricle area was 0.41 ± 0.09. The mean cardiothoracic ratio (CTR) was 0.37 ± 0.07. Some fetal ultrasound images of the cases are presented in Figure 2.

### 3.3. Cardiac geometry

The mean *z*-score value of LV end-diastolic volume was 4.09 ± 0.91 in all left VO cases. According to the location, LVA/D were divided into two subgroups: group A (apical) and group B (free-wall). Compared to the control group, the mean 4CV GSI of group A was significantly higher than that of group B (1.33 ± 0.14 vs. 1.07 ± 0.39, *p *< 0.01), with no significance in the control group (1.33 ± 0.14 vs. 1.29 ± 0.87, *p* = 0.317).

**Figure 3:**
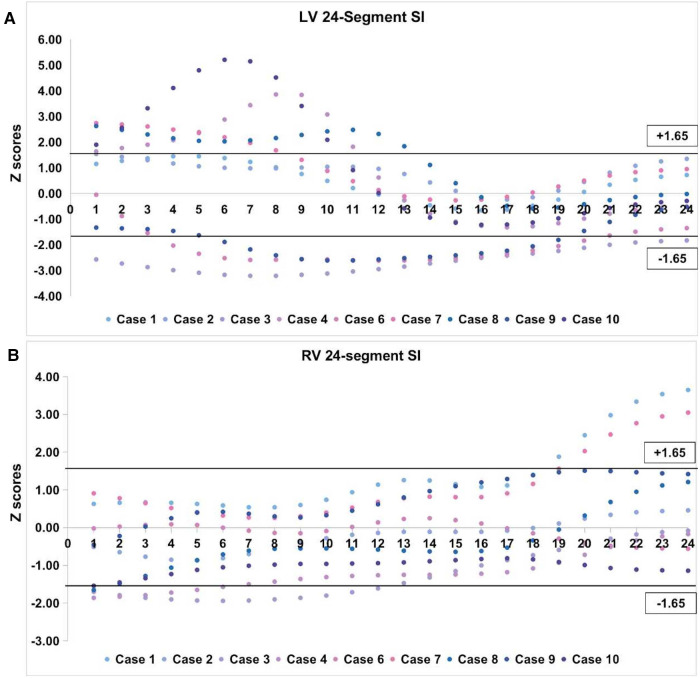
*z*-score values of 24-segment SI in left VO cases. (**A**) Scatter diagram of LV 24-segment SI; (**B**) Scatter diagram of RV 24-segment SI. The black horizontal line above the abscissa represents *z*-score of +1.65 (95th centile) and the one under the abscissa represents *z*-score of −1.65 (5th centile). VO, ventricular outpouching; LV, left ventricle; RV, right ventricle; SI, sphericity index.

For measurements of the 24-segment SI, the intraobserver variability (ICC) was 0.96. The 24-segment SI *z*-score values of both ventricles are shown in a scatter diagram (Figure 3). Four cases had >95th centile SI of LV base segments 1–8 and three had <5th centile segments 1–8. Three cases were <5th centile in LV middle segments 9–16 and three cases had >95th centile SI in part of the LV middle segments 9–16. For LV apical segments, three cases had <5th centile SI values. Two cases had a <5th centile SI value in RV base segments 1–8 and two cases had a >95th centile SI in RV apical segments 17–24.

For the case of RVA, the global width of 4CV was 30.77 mm (*z*-score = 2.03, >95th centile), and the GSI was 1.06 (*z*-score = −1.82, <5th centile). None of the left ventricular SI *z*-score was <5th centile nor over >95th centile. Right ventricular SI *z*-scores were <5th centile for segments 6–12 and <10th centile for segments 4, 5, 13, and 14.

### 3.4. Global contractility

Compared to the control group, the LV GLS of VD/VA was significantly lower (−10.6 ± 3.4 vs. −20.38 ± 5.2, *p *< 0.01). The LV EF and LV FAC of the VD/VA cases were also significantly decreased than the control group (33.37 ± 8.22 vs. 55.84 ± 10.51, *p *< 0.01; and 22.40 ± 6.00 vs. 41.34 ± 8.58, *p *< 0.01, respectively). The mean *z*-score value of LV CO obtained from the fetal HQ was 0.3 ± 0.31, and none was <−1.65. The RV GLS and FAC of LVA/VD and control group were without significant (−18.83 ± 6.03 vs. −18.02 ± 6.16, *p* = 0.718; and 37.08 ± 9.26 vs. 32.60 ± 10.67, *p* = 0.243, respectively). For the RVA fetus, the LV GLS was −16.04% and the RV GS was −11.2%. The FAC of LV and RV were 37.26% and 24.39% (<5th centile), respectively. The computed LVEF was 51.89%.

### 3.5. 24-segment transverse contractility

**Figure 4:**
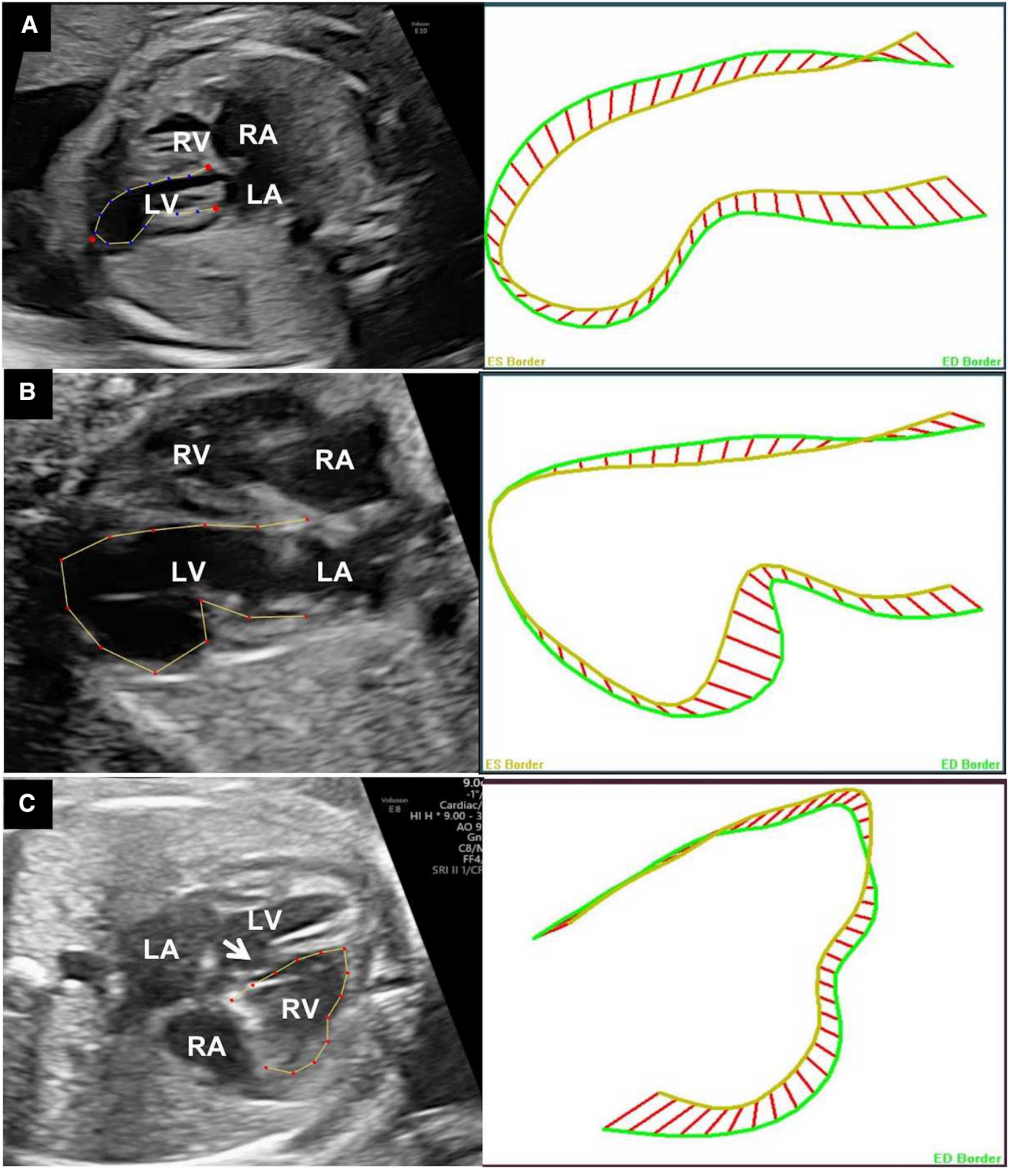
Outlines of the end-systolic and end-diastolic ventricular endocardium of different VOs: apical LVD (**A**), apical LVA (**B**), and RVA of the free-wall (**C**). The white arrow pointing the ventricle septal defect. LA, left atrium; LV, left ventricle; RA, right atrium; RV, right ventricle. VO, ventricular outpouching; LVD, left ventricular diverticulum; RVA, right ventricular aneurysm.

The ICC for the measurements of the 24-segment FS was 0.65. Figure 4 presented the 24-segment outlines of the end-systolic and end-diastolic ventricular endocardium in different kinds of VOs. The global LV contractility was all significantly decreased in the cases. The 24-segment transverse FS was calculated and divided into two groups: affected segments and normal myocardium segments. The mean *z*-score value of the affected segments was significantly lower than the other segments (−2.43 ± 0.70 vs. −1.71 ± 0.92, *p *< 0.01). For the RVA case, almost all segments of RV were <5th centile, and the LV 24 segments FS were within the normal range.

### 3.6. Follow-up

Four cases (4/10, 40%) chose to terminate the pregnancy after fetal echocardiography and consultation, including three LVA cases and the RVA case. Postnatal follow-up was available for all survivors; the median follow-up was 16.5 months, ranging from 1 to 75 months. One case (case 1) received surgical resection of the diverticulum at 5 years old. According to the last measurement before surgery, the area of the diverticula was 680 mm^2^, and the ratio to the area of LV was 0.4, with no significant differences from the previous examination. One case (case 7) was found to be decreased in size according to the last follow-up evaluation, and the other five cases showed a stable area ratio during the follow-up. For the two fetuses associated with arrhythmia, one returned to sinus rhythm soon after birth.

## 4. Discussion

Here, we presented 10 prenatally diagnosed with ventricular aneurysm or diverticulum. We summarized the prenatal characteristics and outcomes of these fetuses. Moreover, we used fetal HQ to evaluate the shape and contractility of the cases. To the best of our knowledge, the present study was innovative in the comprehensive assessment of the morphology and ventricular function using fetal HQ in a series of fetal VO cases.

The prenatal diagnosis of VD and VA is crucial for prenatal consulting and perinatal management (4). The disease is rare, lacks experience in numerous aspects, and is only reported in case series and reviews. In a review study including 809 cases since 1816, only 34 cases (4.2%, 21 LVA and 13 LVD) were diagnosed prenatally (3). Here, we reported 10 prenatal diagnosed cases through 7 years and follow-up afterbirth with a longest of 75 months. The prenatal diagnosis of right VOs has been more infrequent (10), and we described one case in the present study.

The differential diagnosis of VA and VD is not that definitive. The main point widely accepted is that VDs have complete myocardial tissue histologically, while VAs contain fibrous tissue without myocardium. VA was reported to have a significantly poor prognosis as compared to VD. Only 30% of VA patients were alive at 4 years in the case series of Marijon et al. (11). VAs are usually isolated anomalies, and VDs may be associated with anomalies with the midline structures of the body (12). It is much more challenging to make accurate diagnoses prenatally. In our case series, the case of RVA was associated with a perimembranous VSD, and all left VOs were isolated lesions. Early-diagnosed VAs may have a poor prognosis and needs further close follow-up.

The morphological anomaly is the main characteristic of VOs that help make the diagnosis to some extent. The sphericity index is a new index to evaluate the shape of the ventricular chambers and is obtained by calculating the ratio of end-diastolic length diameter and transverse diameter (8). SI changes with different fetal disease states and is independent of gestational age and fetal biometric measurements (13). In our cases, SI changes sensitively to chamber shape changes of all VOs. The GSI and 24-segment SI values of cases with free-wall located VOs were markedly decreased, indicating the abnormally increased transverse width diameters. Moreover, 24-segment SI has the potential to accurately locate the lesion segments according to our application in aneurysm and diverticula cases.

The assessment of fetal heart function is both meaningful and challenging. Ventricular aneurysms and part of ventricular diverticulum are reported to have low contractility in lesion segments (6). The measurement of FS alone from two-dimensional ultrasound is far from illustrating the cardiac function. Previous studies focus on the different contractilities between VA and VD, and less measurement of the ventricular function of both ventricles. In the present study, both global contractility and segment fraction data were obtained and compared with the control group.

Fetal HQ integrates the technique of two-dimensional speckle-tracking echocardiography (2D-STE) and obtains the GLS of both ventricles after automatic measurement. GLS is the fractional percentage of the cardiac wall change and has been shown to be reproducible and angle independent in the fetus (14). Several studies reported an increase in GLS value in both left and right ventricles throughout gestation (15), and some provided stable values during the fetal period (16). The discrepancies in normal GLS values may due to different equipment and ethnic differences. Here, we compared the cases with the normal control group from our center to avoid these differences. As a result, the LV GLS, EF, and FAC of left VOs significantly decreased. While the LV CO of all cases was still at normal ranges, it may be due to the enlarged ventricular chambers considering that the values of LV end-diastolic volume were all over the 95th centile. The contractility measurements of RV were consistent with the control group and even somewhat higher in the data.

Segmental contractility is essential in fetal VOs. The transverse fractional shortening of 24 segments of the right and left ventricles provides a comprehensive method to examine the contractility of the ventricular chambers (9). Though the global contractility was already lower, when compared with normal segments of the same ventricle, the transverse FS was significantly decreased in lesion segments. The results indicated that 24-segment FS could identify the lesion segments sensitively.

Limitations of the present study existed in several aspects. First, the study was done in a single center, and the number of cases was limited to illustrate the prenatal evaluation of VD/VA fully. Moreover, histological data were unavailable for the cases, and the value of fetal HQ in the differential diagnoses of VD and VA is underway in our further study.

## 5. Conclusion

In conclusion, 10 prenatally diagnosed VOs and 30 controls were described in the present study. Fetal HQ was an applicable technique that was easy to operate and obtain valuable data. 24-segment SI has the potential to locate the lesion segments of VOs accurately. Global and segmental contractility were significantly decreased in both VA and VD cases using fetal HQ.

## Data Availability

The original contributions presented in the study are included in the article, further inquiries can be directed to the corresponding authors.
